# Overexpression of activated CaMKII in the CA1 hippocampus impairs context discrimination, but not contextual conditioning

**DOI:** 10.1186/s13041-019-0454-3

**Published:** 2019-04-05

**Authors:** Sanghyun Ye, Ji-il Kim, Jooyoung Kim, Bong-Kiun Kaang

**Affiliations:** School of Biological Sciences, Seoul National University, Gwanak-gu, Seoul, 08826 South Korea

**Keywords:** Context discrimination, CaMKII, Hippocampus, Memory, Contextual fear conditioning

## Abstract

Calcium/Calmodulin-dependent protein kinase II (CaMKII) plays a key role in the molecular mechanism of memory formation. CaMKII is known to be activated specifically in the activated spines during memory formation. However, it is unclear whether the specific activation of CaMKII is necessary for encoding information. Here, we overexpressed active form of CaMKII (CaMKII*) in the hippocampal CA1 region to activate CaMKII nonspecifically. Moreover, we examined context-discrimination performance of mice. We found that the mice with overexpression of CaMKII* showed impaired context-discrimination ability, while the contextual fear conditioning remained intact. These results indicate that spatial specificity of CaMKII activation is necessary for context discrimination.

**Electronic supplementary material:**

The online version of this article (10.1186/s13041-019-0454-3) contains supplementary material, which is available to authorized users.

## Main text

The molecular mechanism of learning and memory has been the subject of numerous neuroscience studies, since decades. Previous studies have revealed that Calcium/Calmodulin-dependent protein kinase II (CaMKII) is activated during memory formation, and initiates biochemical cascades in response to various stimuli in the dendritic spines [1–3]. In addition, it has been reported that CaMKII is specifically activated in the dendritic spines, which in turn are activated by stimuli, to induce the spine maturation [4, 5].

However, there is no evidence about whether the non-specific CaMKII activation perturbs memory formation. Previous studies have shown that overexpression of activated CaMKII (containing T286D, T305A, T306A; CaMKII*) increases spine volume and perturbs memory trace during the maintenance phase [6, 7]. Therefore, selective activation of CaMKII in specific spines might be necessary for intact memory formation. To test this, we overexpressed activated CaMKII in the hippocampal CA1 region, which is necessary for memory formation [8].

We expressed the active form of CaMKII (CaMKII*) or mCherry under the CaMKII-alpha promoter in the human embryonic kidney (HEK) 293 T cells, which was delivered by adeno-associated virus (AAV) [9]. We confirmed successful overexpression in the HEK 293 T cells (Additional file 1: Figure S1a). To evaluate the expression in the CA1 hippocampus, we used human influenza hemagglutinin (HA) tag, and confirmed HA expression in the hippocampal CA1 region slice after performing a behavioral test (Additional file 1: Figure S1a and b).

**Fig. 1:**
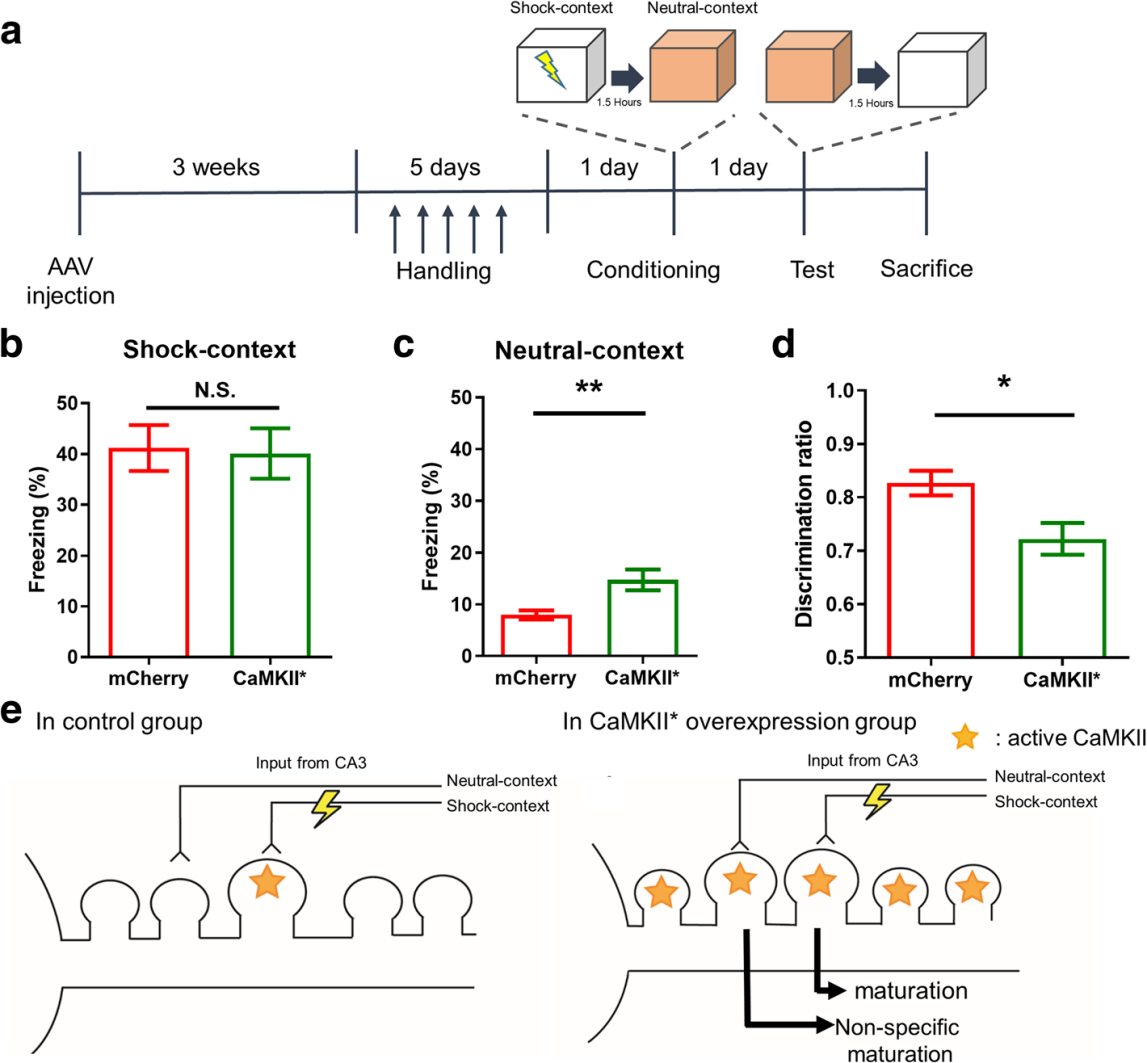
Overexpression of CaMKII* in hippocampal CA1 region impairs context discrimination but not contextual fear conditioning. **a** schematic showing the experimental protocol (**b**) Mean percentage of time spent in freezing after 1 day of conditioning in shock context (*n* = 12 for control group, and *n* = 13 for overexpression group). **c** Mean percentage of time spent in freezing after 1 day of contextual conditioning in neutral context (*n* = 12 for control group, and *n* = 13 for overexpression group). **d** Mean percentage of discriminative ratio, which is calculated using freezing level in the shock context divided by (shock context freezing + neutral context freezing) (*n* = 12 for control group, and *n* = 13 for overexpression group). All graphs show mean ± SEM. Student’s t-test. N.S., not significant; *P < 0.05; **P < 0.01 versus control group. **e** Schematic showing the effect of CaMKII* overexpression in specific spine maturation during memory formation in the control and overexpression groups

After the injection of viruses in the hippocampal CA1 region, we examined the effect of CaMKII* overexpression via evaluation of context discrimination, which is a hippocampus-dependent form of associative learning [10, 11]. To quantify the context-discrimination ability, mice were trained to discriminate between two contexts, namely shock and neutral contexts. In the shock-context chamber, mice received a shock; while in the neutral-context chamber, they just explored. We examined the percentage of the time spent freezing (freezing level) after 1 day of conditioning in each context (Fig. 1a).

We found that both the groups of mice showed a similar freezing level in the shock context. This indicates that CaMKII* overexpression did not affect the contextual fear conditioning (Fig. 1b). However, in the neutral context, the mice with CaMKII* overexpression showed an increased freezing level (Fig. 1c). This implies that the ability of mice to discriminate different contexts is decreased by CaMKII* overexpression. In addition, CaMKII* group showed significantly higher discriminative ratio (Fig. 1d) [12].

Moreover, we examined the freezing level in shock-context chamber after 3 weeks of the conditioning to assess remote memory. The mice showed similar freezing levels both after 1 day and 3 weeks of conditioning, and there was no difference between the two groups (Additional file 1: Figure S2). These results show that CaMKII* overexpression does not impair the ability of mice to maintain contextual memory.

The present study demonstrates that CaMKII* overexpression in the hippocampal CA1 region can impair the ability of context discrimination; however, the contextual fear conditioning remains intact. One possible explanation for this may include specificity of spine maturation. In a normal state, CaMKII activation may only strengthen the synapses between engram cells during memory formation (Fig. 1e) [13]. However, when CaMKII* is overexpressed, it may not only activate specific synapses, but also other synapses (Fig. 1e). A previous study has shown that CaMKII* overexpression can produce synaptic potentiation and increase spine volume, which supports this explanation [6]. In addition, a previous study has shown that hippocampal lesion may impair the ability of context discrimination, but not fear conditioning [10]. Thus, it might be possible that the impairment of context-discrimination ability in this study was due to malfunction of the hippocampus.

Loss of context-discrimination ability is also related to post-traumatic stress disorder (PTSD) [14]. Impairment of context discrimination in the mice with CaMKII* overexpression implies that excessive CaMKII activation may be responsible for this disorder. Therefore, further studies are required to reveal the relationship between CaMKII activity, spine specificity, and memory discrimination. This may help to better understand the disorder to find its cure.

## Additional files

Additional file 1: **Material and Methods**. **Figure S1.** Validation of CaMKII overexpression in the HEK 293 T cells and the hippocampal CA1 region. **Figure S2.** Remote-memory retrieval test in shock paired context. (DOCX 1325 kb)

## Abbreviations

CaMKII: Ca2+/calmodulin-dependent kinase II
CaMKII*: Active form of Ca2+/calmodulin-dependent kinase II (containing T286D, T305A, T306A mutations)
HA: Human influenza hemagglutinin
HEK: Human embryonic kidney
PTSD: Post-traumatic stress disorder
