# Supplementary material for: Overexpression of activated CaMKII in the CA1 hippocampus impairs context discrimination, but not contextual conditioning
Source: Mol Brain. 2019 Apr 5;12:32. doi: 10.1186/s13041-019-0454-3 (PMC6449978; doi:10.1186/s13041-019-0454-3)
Supplement: Supplementary file 1 — Material and Methods. Figure S1. Validation of CaMKII overexpression in the HEK 293 T cells and the hippocampal CA1 region. Figure S2. Remote-memory retrieval test in shock paired context. (DOCX 1325 kb) [file 13041_2019_454_MOESM1_ESM.docx]

**Additional files for**

**Title:** Overexpression of activated CaMKII in the CA1 hippocampus impairs context discrimination, but not contextual conditioning

**Authors:** Sanghyun Ye^1^, Ji-il Kim^1^, Jooyoung Kim^1^, Bong-Kiun Kaang^1^

**Material and Methods**

**Animals**

8~12 weeks old male C57BL/6N mice were used in all experiments. All mice were raised under a 12-hr light/dark cycle and given food and water by ad libitum. The behavioral experiments were performed during the light cycle. All procedures were conducted according as the regulation and guidelines from the Institutional Animal Care and Use Committee (IACUC) of Seoul National University

**AAV production and stereotactic viral injection**

Adeno-Associated Viruses were produced with both serotype 1 and 2 capsids (AAV 1/2) using HEK293T cells. The titer was measured by quantitative real-time PCR (Prism 7300, Applied Biosystems, USA). Mice were anaesthetized with a ketamine/xylazine mixture and arranged in a stereotaxic apparatus (Stoelting Co. USA). The hippocampal CA1 (AP: -1.8 mm/ ML: ±1.5 mm/ DV: -1.65 mm below the skull surface) was targeted, and the viruses (HA-tagged CaMKII* or mCherry) were delivered bilaterally by 33 gauge needle with Hamilton syringe at a rate of 0.1 μl/min. After injection, the needle stayed in the target region for an additional 6 minutes and was withdrawn. AAV 1/2s were expressed for 3 weeks.

**Context discrimination test**

All mice were conditioned 3 weeks after the AAV injection. Mice were habituated to the hands of the investigator on 5 consecutive days and were trained in two different contexts: shock-context and neutral-context. The mice were exposed square chamber with a steel grid (Med Association Inc., St Albans, VT) during the 3 min and 0.5mA shock of 2 s duration was delivered at 148s. After 1.5 hours, the mice were exposed to the different chamber containing stripe wallpaper and different grid during the 3 min. Next day or after 3 weeks, the mice were exposed in the neutral-context and shock-context using 1.5 hours interval between two context exposures. Time percentage of freezing was measured during the 3 min at each chambers.

**Western blot analysis**

The hippocampal CA1 region or HEK293T cell was homogenized with lysis buffer (50 mM pH 7.6 Tris-Cl, 150 mM NaCl, 1 mM EDTA, 1% NP-40, 0.1% SDS, 1mM DTT, 0.5% sodium deoxycholate) containing protease inhibitor cocktail. Electrophoresis of equal amounts of protein was conducted on 4-12% SDS-polyacrylamide gels (Invitrogen, USA). Samples were analyzed by western blot using the following antibodies: anti-GAPDH (Ambion), anti-CaMKII (Abcam) and anti-HA (Santacruz)

**Immunohistochemistry and fluorescence imaging**

Perfused brains were fixed in 4% paraformaldehyde in PBS and dehydrated by 30% sucrose in PBS. Brains were sliced into 35μm cryosectioning (Leica Ltd., Germany), and slices for mCherry were mounted in VECTASHIELD medium containing DAPI. Brain slices for HA-tagged CaMKII* were stained with anti-HA antibody (Roche, 1:1,000) and goat anti-rabbit Alexa Fluor 488 IgG (Invitrogen, 1:500). Brain slice were imaged by Leica SP8 confocal microscope.

**Supplementary Figures and Figures legends**

**Figure S1. Validation of CaMKII overexpression in the HEK293T cell and the hippocampal CA1 region.**


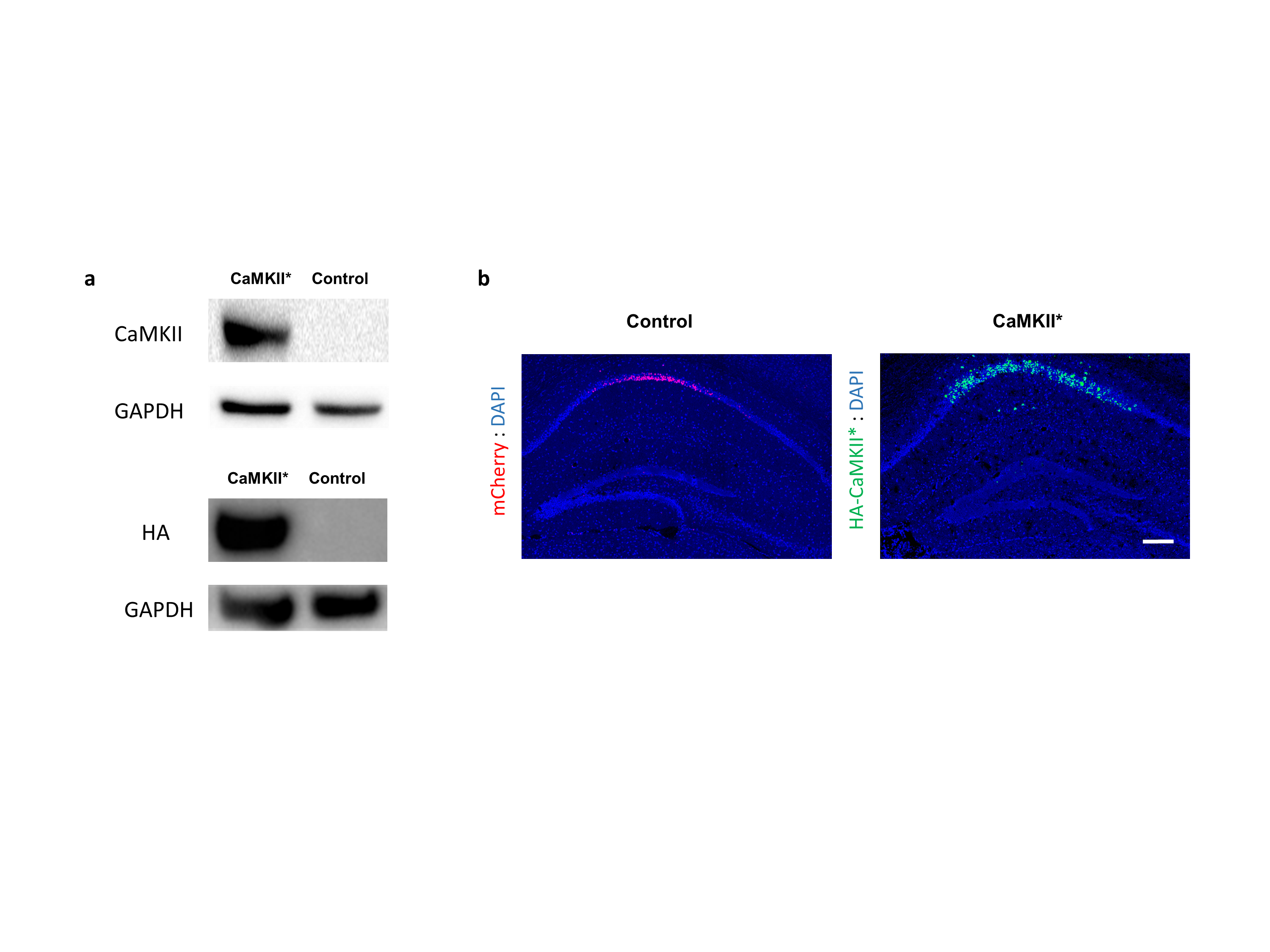


(a) Results showing overexpressed CaMKII in HEK293T cell (upper panel) and the hippocampal CA1 region (lower panel). (b) Representative images of mCherry or HA-tagged CaMKII* in the hippocampal CA1 region (Blue: DAPI, Red: mCherry, Green: HA-tagged CaMKII*). Left panel is control group and right panel is CaMKII* group. Scale bar represents 200μm.

**Figure S2. Remote-memory retrieval test in shock paired context.**


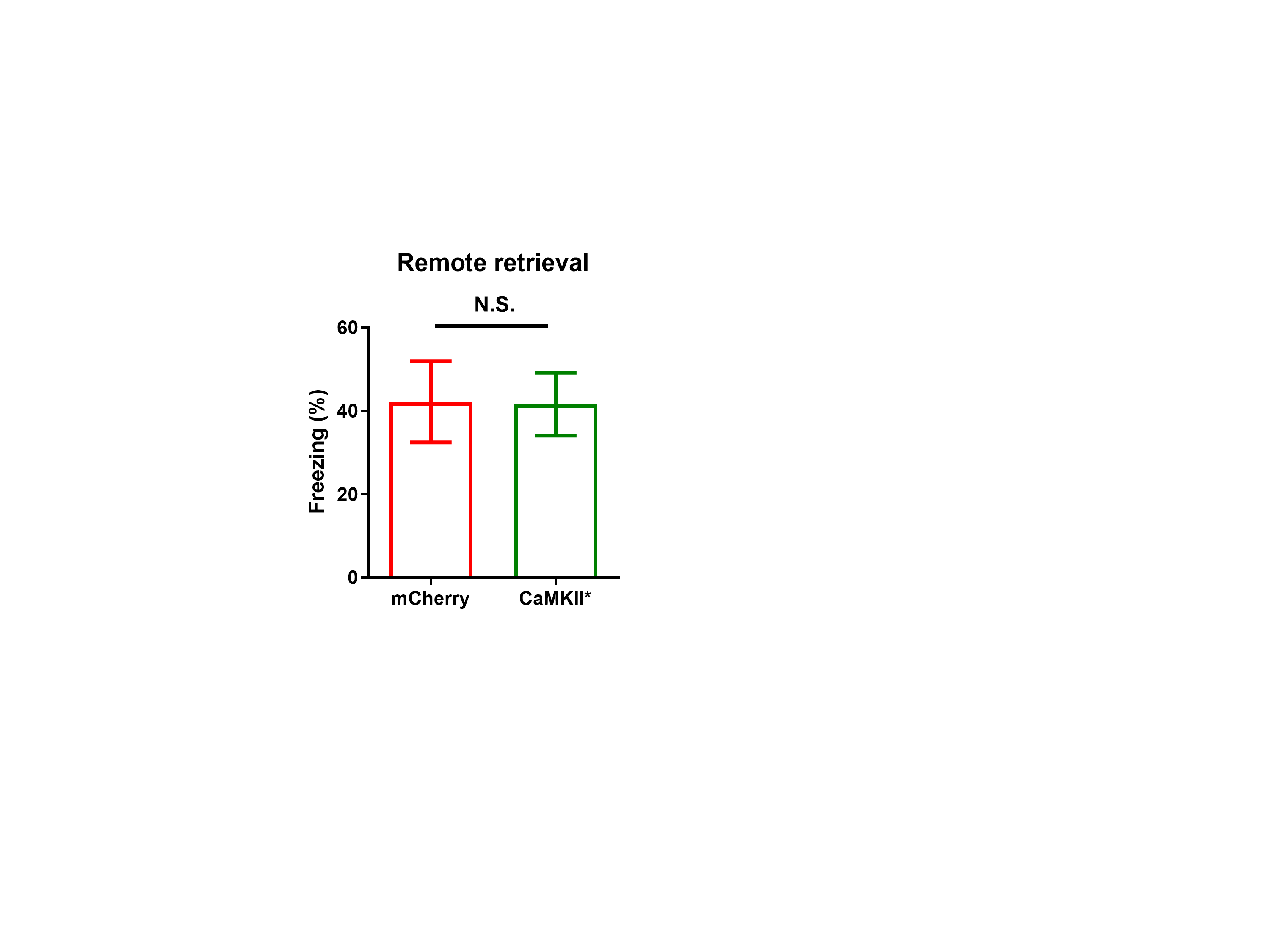


Mean percentage of time spent in freezing 3 weeks after contextual conditioning in shock paired context (n=5 for control group, and n=5 for overexpression group). graphs show mean ± SEM. Student’s *t*-test. N.S., not significant.
